# Broad-linewidth sources result in a skin-tone bias in noninvasive optical measurement of oxygen saturation

**DOI:** 10.1117/1.JBO.31.7.070501

**Published:** 2026-07-09

**Authors:** Giles Blaney, Sergio Fantini

**Affiliations:** Tufts University, Department of Biomedical Engineering, Medford, Massachusetts, United States

**Keywords:** pulse oximetry, skin tone, melanin, oxygen saturation, polychromatic source, monochromatic source

## Abstract

**Significance:**

Pulse oximeters overestimate arterial oxygen saturation in patients with darker skin tones, potentially increasing rates of occult hypoxemia. The emission of broad-linewidth light-emitting diodes (LEDs) has been proposed as a possible cause for this melanin-dependent bias.

**Aim:**

This letter utilizes a physics-based model to investigate if the spectral bandwidth of light sources contributes to melanin-dependent measurement biases.

**Approach:**

Pulse oximetry measurements were simulated across varying melanin concentrations and source bandwidths using a hybrid diffusion theory and Monte Carlo model. Epidermal melanin absorption was modeled with transmissive filters on the surfaces of an infinite slab representing bulk tissue.

**Results:**

Simulations demonstrate that pulse oximeter calibration curves depend on melanin concentration when broad-linewidth LEDs are utilized. Conversely, calibration curves remain independent of melanin when employing monochromatic laser diodes (LDs).

**Conclusion:**

Spectral coloring from broad-linewidth LEDs contributes to the skin-tone bias observed in pulse oximetry. Although updating calibration methods for different skin tones is a potential approach, adopting monochromatic LDs could alternatively eliminate this spectral-coloring contribution to the bias.

In recent years, there has been increasing evidence that current pulse oximeter technology—and likely other optical oxygen saturation technologies—exhibit a skin-tone-dependent bias.^1^ Specifically, pulse oximeters overestimate arterial Oxygen Saturation (${\mathrm{SaO}}_{2}$) at a higher rate for Black versus White patients. This problem has the potential to have significant public health impacts by increasing rates of occult hypoxemia for patients with darker skin tone.^2^ A plausible explanation is that the pulse oximeter calibration curves—found via empirical methods—depend on skin tone. Because the United States Food and Drug Administration (US FDA) requires calibration to be performed on a population including 15% dark-skin subjects,^3^ it is reasonable to assume that the resulting calibration curves are biased toward those of lighter-skinned people. Given the skin-tone dependence of the oxygen saturation readings of LED-based pulse oximeters that rely on preliminary measurements in a calibration cohort, the skin-tone distribution in the calibration cohort will determine the nature of the skin-tone bias. However, the physical origin of differing calibration curves and thus the bias remains unclear. Understanding it will help develop future methods to reduce or eliminate this bias. The purpose of this work is to utilize a physics-based model to test if a melanin-concentration-dependent bias can be attributed to broad-linewidth light sources, which has been proposed as an origin of this bias.^4^^,^^5^

Previous physics-based models have simulated the pulse oximetry measurement considering skin tone with Monte Carlo simulations,^6^^,^^7^ but to our knowledge, no simulation has been presented that specifically considers the spectral bandwidth of the light source. Our previous Monte Carlo model focused on the development of a measurement type insensitive to absorption changes in superficial tissue.^7^ In that work, we hypothesized that this measurement would alleviate skin tone-related effects due to the inherent insensitivity to melanated tissue. Al-Halawani et al. developed a similar Monte Carlo model and simulated the conventional pulse oximeter calibration procedure, showing different calibration curves for different skin tones, which could explain the observed skin-tone bias.^6^ However, from these works, the physical origin of the bias remained unclear, making it difficult to recommend directions for technology development.

In Ref. 4, Bierman et al. proposed spectral coloring^8^ of the broad-linewidth light emitting diodes (LEDs) as the reason for the melanin-dependent bias. Their work experimentally showed—on 31 subjects—that the measured Ratio-of-ratios (R)—the key measured parameter in pulse oximetry—depended on the choice of broad-linewidth versus monochromatic light sources.^4^ Importantly, the strength of the dependence of R on light source type depended on melanin volume fraction (M), which would explain a skin-tone bias arising from different calibration curves for different skin tones. This led to the recommendation that monochromatic laser diodes (LDs) be used instead of broadband LEDs.^2^

The work of Al-Halawani et al.^6^ claimed to explain the skin-tone bias without considering broad-linewidth sources, whereas Bierman et al.^4^ suggested that broad-linewidth sources are necessary to create this bias. This inconsistency may be due to choices in the Monte Carlo model, such as attributing the pulsatile signal to a change in the entire blood volume in the dermis—including venous blood—or the choice to model a wavelength-dependent absorption background separate from melanin, which also depended on blood volume.^6^ Multiple effects could cause the clinically observed skin-tone bias,^1^ so that the results in Refs. 4 and 6 may not be inconsistent but rather point to compounding effects. Regardless, work has yet to be done to test the physical explanation of spectral coloring of broad-linewidth sources proposed in Ref. 4 for pulse oximetry. Therefore, in this work, we utilized a physics-based model—based on our work in Refs. 7 and 9—which considered broad-linewidth sources using a hybrid Monte Carlo and diffusion theory model.

We modeled the tissue as an infinite slab using extrapolated boundaries in diffusion theory,^10^ including a transmissive filter on the slab surfaces (Fig. 1). The transmissive filters simulated the absorption of the epidermis according to the following equation for the absorption coefficient ($\mu_{a}$) spectrum [Fig. 1(a)]:^11^

$${\mu_{a}}_{,\mathrm{epi}}(\lambda ,M)=M\times 51.9\;{\mathrm{mm}}^{-1}\times ({\lambda /500\;\mathrm{nm})}^{-3.5},$$ (1)

where M is the melanin volume fraction and λ is the wavelength. The thickness of the transmissive filters—$\ell_{\mathrm{eff}}/2$ in Fig. 1—was modeled as half the partial optical path-length in the epidermis (⟨ℓ⟩/2). The Monte Carlo model in Ref. 7 was used to compute these epidermal partial optical path-lengths for each wavelength (λ) from 550 to 950 nm spaced by 1 nm by launching $3\times 10^{10}$ photons for each wavelength (n.b., for 950 to 1050 nm in this work ⟨ℓ⟩ was linearly extrapolated). The model considers the thickness of the transmissive filters as a function of λ and M (i.e., ⟨ℓ⟩(λ,M)). The example plots in Fig. 1 show an M of 43%—the maximum value considered in this work.

The bulk tissue in between the transmissive filters was modeled with optical properties (Fig. 1(b)) based on volume-weighted averages of the separate finger tissue types found in Ref. 7. Specifically, $\mu_{a}$ was modeled using 66  μM total-hemoglobin concentration, 65% tissue oxygen saturation, 55% water volume fraction, and 16% lipid volume fraction according to Beer’s law. The reduced scattering coefficient ($\mu_{s}^{\prime }$) of the bulk tissue was modeled as a combination of 14% Rayleigh and 86% Mie scattering with a Mie scattering power of −0.7 and $\mu_{s}^{\prime }$ at 500 nm of $2.41\;{\mathrm{mm}}^{-1}$. Again, as in Ref. 7, the refractive indices of the bulk tissue were modeled as a volume-weighted average of the dry refractive index—assumed to be 1.51—and the refractive index of water.

Two types of optical sources were considered, LDs and LEDs, each emitting a power spectral density spectrum ($P_{\mathrm{src}}(\lambda )$) with two peak wavelengths of 660 and 880 nm—inspired by the Analog Devices MAX30102.^12^ LDs were modeled with an ideal infinitesimally small bandwidth so that they only emit at their peak wavelength; LEDs were modeled as a Gaussian lineshape whose width was parameterized with the full width at half maximum (FWHM). The example plots in Fig. 1 consider a LED with 660 nm peak and 50 nm FWHM. Figure 1(d) shows an example of the spectral coloring effect, where the spectrum of the detected light is effectively red-shifted by the melanin absorption and the tissue optical properties. Importantly, this shift is dependent on M, and this effect is the key focus of this work.

**Fig. 1:**
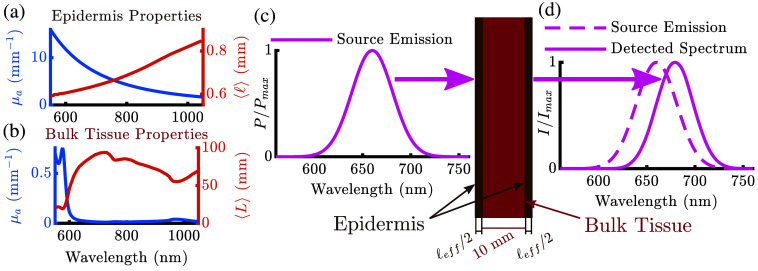
Pulse oximetry model considered in this work. Example subplots considering 43% melanin volume fraction and 50 nm full width at half maximum. (a) Epidermal optical properties with partial optical path-length computed from Monte Carlo. (b) Bulk tissue optical properties for the diffusion theory slab model. (c) Normalized source emission power spectral density. (d) Spectrally colored detected intensity spectrum compared with source emission.

To simulate the pulsatile signal measured in pulse oximetry, a 1  μM perturbation in total-hemoglobin concentration (Δ[HbT]) was modeled with oxygen saturation determined by the modeled ${\mathrm{SaO}}_{2}$ (i.e., $\Delta [{\mathrm{HbO}}_{2}]={\mathrm{SaO}}_{2}\times \Delta [\mathrm{HbT}]$ and $\Delta [\mathrm{Hb}]=(1-{\mathrm{SaO}}_{2})\times \Delta [\mathrm{HbT}]$). The perturbation in $\mu_{a}$ (i.e., $\Delta \mu_{a}(\lambda )$) from $\Delta [{\mathrm{HbO}}_{2}]$ and Δ[Hb] was then computed using Beer’s law. The detectors were modeled with a constant efficiency across wavelength—a real photodiode’s wavelength-dependent responsivity would act as an additional spectral factor on the source term in Eqs. (2) and (3), contributing to the same spectral coloring—thus the detected signal ($I_{\det}$) is considered the integral of the transmitted intensity spectrum across λ as follows:

$$I_{\det,\mathrm{sys}}({\mathrm{SaO}}_{2},M)=\int P_{\mathrm{src}}(\lambda )\times e^{-{\mu_{a}}_{,\mathrm{epi}}(\lambda ,M)\times \ell_{\mathrm{eff}}(\lambda ,M)}\;\times T_{\mathrm{Grn}}({\mu_{a}}_{,\mathrm{tis}}(\lambda )+\Delta \mu_{a}(\lambda ,{\mathrm{SaO}}_{2})/2,{\mu_{s}^{\prime }}_{,\mathrm{tis}}(\lambda ))d\lambda ,$$ (2)

$$I_{\det,\mathrm{dia}}({\mathrm{SaO}}_{2},M)=\int P_{\mathrm{src}}(\lambda )\times e^{-{\mu_{a}}_{,\mathrm{epi}}(\lambda ,M)\times \ell_{\mathrm{eff}}(\lambda ,M)}\;\times T_{\mathrm{Grn}}({\mu_{a}}_{,\mathrm{tis}}(\lambda )-\Delta \mu_{a}(\lambda ,{\mathrm{SaO}}_{2})/2,{\mu_{s}^{\prime }}_{,\mathrm{tis}}(\lambda ))d\lambda ,$$ (3)

where the first term in the integral accounts for the source power spectral density ($P_{\mathrm{src}}$), the second for the epidermal transmissive filters, and the third is the Green’s function of transmittance for the bulk tissue ($T_{\mathrm{Grn}}$) considering a pencil beam illumination.^10^ The two detected intensities, $I_{\det,\mathrm{sys}}$ and $I_{\det,\mathrm{dia}}$, correspond to the systolic and diastolic phases, respectively. These values were computed for each nominal center wavelength and FWHM, resulting in different values for the 660 and 880 nm sources (e.g., $[I_{\det,\mathrm{sys}}{]}_{660\;\mathrm{nm}}$) and the light source type (e.g., LED).

To recover ${\mathrm{SaO}}_{2}$ from the detected intensities, the method in Ref. 9—based on the modified Beer-Lambert law—was employed. This method can be summarized with the following equation:

$${\mathrm{SaO}}_{2}=\frac{-\varepsilon_{[\mathrm{Hb}],660\;\mathrm{nm}}+\varepsilon_{[\mathrm{Hb}],880\;\mathrm{nm}}(\frac{\langle L\rangle_{880\;\mathrm{nm}}}{\langle L\rangle_{660\;\mathrm{nm}}})R}{(\varepsilon_{[{\mathrm{HbO}}_{2}],660\;\mathrm{nm}}-\varepsilon_{[\mathrm{Hb}],660\;\mathrm{nm}})+(\varepsilon_{[\mathrm{Hb}],880\;\mathrm{nm}}-\varepsilon_{[{\mathrm{HbO}}_{2}],880\;\mathrm{nm}})(\frac{\langle L\rangle_{880\;\mathrm{nm}}}{\langle L\rangle_{660\;\mathrm{nm}}})R},$$ (4)

where the R is defined as

$$R=\frac{{\left[\left(I_{\det,\mathrm{sys}}-I_{\det,\mathrm{dia}}\right)/I_{\det,\mathrm{dia}}\right]}_{660\;\mathrm{nm}}}{{\left[\left(I_{\det,\mathrm{sys}}-I_{\det,\mathrm{dia}}\right)/I_{\det,\mathrm{dia}}\right]}_{880\;\mathrm{nm}}}.$$ (5)

This method relies on knowledge of the optical path lengths in tissue (⟨L⟩s) and the extinction coefficients (ϵs)—of both oxy- and deoxy-hemoglobin—at the red and infrared wavelengths (i.e., 660 and 880 nm, respectively). These coefficients are typically found with empirical calibration in conventional pulse oximetry. Instead, for the ideal system in this work, we initially consider the ⟨L⟩s and ϵs for the nominal source wavelengths of 660 and 880 nm. The ⟨L⟩s for bulk tissue (Fig. 1(b)) were found by implementing $\langle L\rangle =-d\ln(T_{\mathrm{Grn}})/d\mu_{a}$ numerically (n.b., $d\mu_{a}=1\times 10^{-6}\;{\mathrm{mm}}^{-1}$). Later, we will attempt to correct for the spectral linewidth by modifying the ϵs, but do not attempt to modify the ⟨L⟩s since it is difficult to find their spectra experimentally.

Figures 2(a) and 2(b) show the pulse oximetry calibration curves, while Figs. 2(c) and 2(d) show the slopes of these calibration curves as a function of M. Figures 2(a) and 2(c) consider LDs, while Figs. 2(b) and 2(d) consider LEDs with a FWHM of 50 nm. The most notable result is that the calibration curve does not depend on M for LDs but does for LEDs. This is due to the spectral shift—seen in Fig. 1(d)—that depends on M. Now—for broad-linewidth sources—the correct coefficients in Eq. (4) no longer belong to the nominal λs of 660 and 880 nm, because the central wavelength of the transmitted spectrum depends on M; their wavelength dependence over the detected band must also be considered. Fundamentally, this bias arises in the application of Beer’s law—the step that converts the pulsatile absorption coefficient changes to the ratio of chromophore concentration changes defining saturation—because the appropriate chromophore extinction coefficients depend on the melanin-shifted detected spectrum and are unknown a priori. The steepening of the slope for increasing M is consistent with the effect seen in Ref. 6, suggesting that spectral coloring from broad-linewidth sources may compound the bias observed in that model—which did not consider broad-linewidth sources. In addition, the slope for the case with no melanin (i.e., M=0) changes when LEDs are considered compared to LDs. This reflects the wavelength dependence of the bulk tissue’s own optical properties; however, empirical calibration may correct for it if the Green’s function spectrum is relatively constant amongst people. These results suggest that the pulse oximetry calibration curve depends on skin tone if broad-linewidth sources—such as LEDs—are employed, but the effect goes away when considering monochromatic sources—such as LDs.

**Fig. 2:**
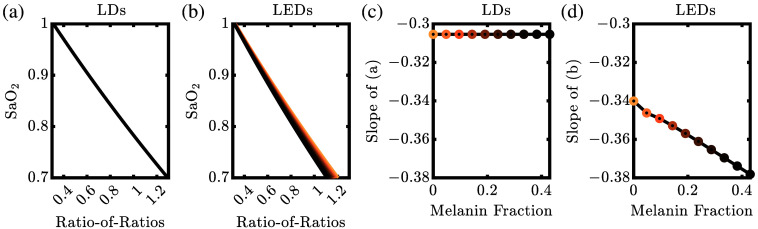
(a), (b) Simulated pulse oximetry calibration curves; (c), (d) calibration curve slopes. FWHM of 50 nm is considered in (b) and (d).

Figure 3 quantifies the error between recovered and modeled ${\mathrm{SaO}}_{2}$ from Eq. (4), where assumed ϵs are those at 660 and 880 nm. For LDs [Fig. 3(a)], the nominal εs give perfect recovery with no error. However, the LED case [Fig. 3(b)] shows an increasing error with increasing M and decreasing ${\mathrm{SaO}}_{2}$—here, results for a LED FWHM of 50 nm are plotted. In an attempt to correct for the spectral coloring, we propose updating the ϵs based on the transmitted spectra reaching the detectors. Specifically, implementing Eq. (4) with weighted average ϵs using the spectral contributions to the detected intensity reported in Eqs. (2) and (3) as weights. The results of this correction are shown in Fig. 3(c), which achieves more than a two times reduction in absolute error. However, Fig. 3(c) does not achieve perfect results—as with LDs in Fig. 3(a)—likely due to not updating the ⟨L⟩s in Eq. (4). It is likely that this residual error arises because the optical path lengths, such as the extinction coefficients, should be averaged over the melanin-shifted detected spectrum rather than evaluated at the nominal wavelengths. Correcting the spectral coloring with weighted ⟨L⟩s is challenging since their spectra are not well known for real tissue.

**Fig. 3:**
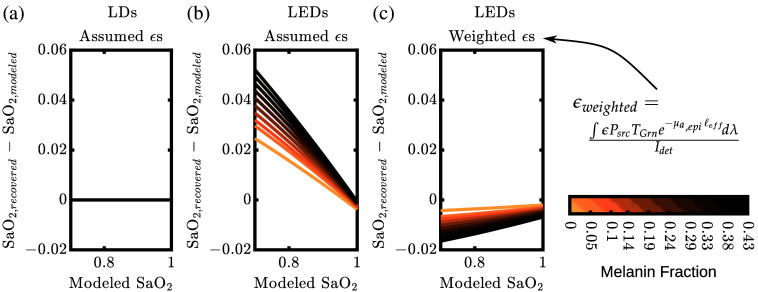
Errors between modeled and recovered ${\mathrm{SaO}}_{2}$. (a), (b) Using assumed extinction coefficients from nominal wavelengths. (c) Using ϵS weighted by detected spectrum. FWHM of 50 nm is considered in (b) and (c).

Figure 4 shows the influence of the sources’ FWHM in terms of the root mean square (RMS) error (n.b., the FWHM of 0 nm is associated with the LDs). As suggested from Fig. 3, a comparison between Figs. 4(a) and 4(b) shows about a two times improvement in error by implementing weighted ϵs. Overall, the error in ${\mathrm{SaO}}_{2}$ increases with increasing FWHM as expected from the spectral coloring effect, and the error is more pronounced for higher M—corresponding to darker skin tones. Figures 1–3 show examples of a 50 nm FWHM—chosen to make the spectral-coloring effect visually clear and to represent the upper end of the expected pulse-oximeter emission bandwidth range, thus Fig. 4 can be used to get a sense of how these figures rescale with different FWHM. Real LEDs’ FWHM can vary significantly depending on the specific type and peak wavelength, with pulse oximeter LEDs roughly between 20 and 50 nm.^4^^,^^12^ We opt to model a larger range because real LEDs emission spectra are not Gaussian and are usually asymmetric with tails decaying less quickly than a Gaussian.^12^ Therefore, these FWHM values may not correspond directly to the FWHMs reported on pulse oximeter data sheets. Instead, these results show that even with this ideal theoretical model, pulse oximetry is expected to have a skin tone (i.e., M) dependent error when broad-linewidth sources are used, and this error increases with increasing source bandwidth.

**Fig. 4:**
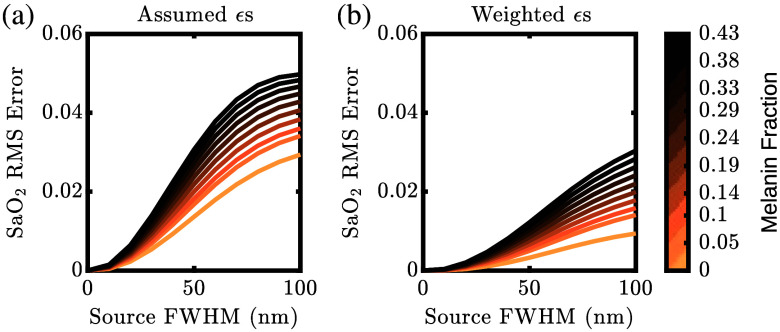
Root mean square (RMS) error of ${\mathrm{SaO}}_{2}$ as a function of source FWHM.

The results in this work show that sources with a broad linewidth can account for the clinically observed skin-tone bias^1^ in pulse oximetry as suggested by Bierman et al.^4^ The direction of the bias is consistent with an overestimation in ${\mathrm{SaO}}_{2}$ for darker skin tones, which would lead to increased rates of occult hypoxemia. Further, the calibration curve slope’s dependence on M was in the same direction as observed by Al-Halawani et al.;^6^ because they did not consider broad-linewidth sources, their bias likely has a different origin, but the matching M-dependence suggests the effects may compound into an even stronger dependence. Overall, these results confirm Bierman et al.’s experimental observation^4^ and point to a different physical origin than Al-Halawani et al.^6^ Our findings are further complemented by Putcha et al.,^13^ who, using a split-pigmentation porcine model, found experimentally that pigmentation attenuates the sensitivity of red-light absorption to oxygen-saturation changes while the near-infrared channel is less affected—consistent with the wavelength-dependent melanin effect identified here.

We base this study primarily on diffusion theory^10^ rather than our previous Monte Carlo model^7^ for two reasons: the diffusion theory model is simpler and easier to interpret, helping draw more concrete conclusions about the physical origin of the bias, and it does not require specialized computer hardware to run (e.g., Ref. 7 utilized a large graphics-card-based Monte Carlo). Importantly, the latter lets us publish the simulation code along with this work for readers to reproduce these results and adopt this approach.^14^

To complement our approach based on diffusion theory, we obtained partial optical path-lengths in the epidermis with our Monte Carlo model.^7^ This is largely because it is impractical to model the epidermis with diffusion theory due to its small thickness and high absorption. In addition, we recomputed the model with constant-thickness melanin filters (i.e., 0.25 mm on each side) which led to similar dependencies on M as those presented, thus not changing our conclusions. We further note that melanin is not dissolved uniformly but resides in discrete melanosomes; the spectrum used here represents the resulting bulk-equivalent epidermal absorption through a melanosome volume fraction,^11^ appropriate for the systematic bias studied here, although this discrete distribution may additionally introduce melanin-associated measurement uncertainty.^2^ A second limitation of the diffusion theory model is the choice to model bulk tissue as a homogeneous slab with a uniform absorption perturbation forming the pulsatile signal. To confirm this does not affect our conclusions, we reproduced the trends in Fig. 2 using our Monte Carlo model.^7^ Therefore, again we do not think our choice of model changes our conclusion that broad-linewidth light sources may be the physical origin of the observed skin-tone bias.

The conclusion that broad-linewidth light sources can cause skin-tone biases—due to the spectral coloring necessitating different extinction coefficients and optical path-lengths to be used for different skin tones—applies to fields beyond pulse oximetry.^8^ Any optical method that relies on wavelength-dependent measurements (i.e., spectroscopy) of tissue noninvasively—including reflectance-mode devices, for which the same integration over a melanin-shifted spectrum applies through a reflectance Green’s function—could fall victim to the skin-tone bias observed here. The issue lies in the integration of an optical spectrum over wavelength by the detector when the centroid of that spectrum depends on melanin concentration. If the detected optical spectrum is effectively a single wavelength (i.e., monochromatic), the light cannot be colored by the tissue, and this consideration is moot. Therefore, we confirm the recommendation of Bierman et al.^2^^,^^4^ to utilize LDs instead of LEDs to eliminate this spectral-coloring contribution to the skin-tone bias in optical measurements of oxygen saturation of tissue. We emphasize, however, that monochromatic sources remove the spectral-coloring mechanism modeled here but need not eliminate other melanin-dependent effects—such as the reduced signal-to-noise ratio that can persist even with laser-diode sources.^15^ Alternatively, the calibration equation used by pulse oximeters could be updated for each skin tone case if broad-linewidth sources must be used—similar to our proposed solution of spectral weighting the extinction coefficients. Intermediate hardware options—such as narrower-band or spectrally filtered LEDs, or additional wavelengths—could likewise reduce this contribution where laser diodes are impractical. However, this would require a separate assessment of skin tone to find the correct calibration curve and would amount to the oximeter analysis being patient-specific, which could introduce further complications. Since, in our opinion, it is preferred to develop an oximetry method that is agnostic to skin tone, we think the better solution is for LD (i.e., monochromatic) based pulse oximeter technology to be pursued considering the errors observed with this model when broadband sources are used.

## Data Availability

Code associated with this work can be found in Ref. 14.
